# Genome-wide association study revealed significant SNPs for anthracnose resistance, seed alkaloids and protein content in white lupin

**DOI:** 10.1007/s00122-024-04665-2

**Published:** 2024-06-10

**Authors:** Grit Schwertfirm, Michael Schneider, Florian Haase, Christine Riedel, Mariateresa Lazzaro, Brigitte Ruge-Wehling, Guenther Schweizer

**Affiliations:** 1 Bavarian State Research Center for Agriculture (LfL), Institute for Crop Science and Plant Breeding, Am Gereuth 2, 85354 Freising, Germany; 2 Department of Crop Sciences, Research Institute of Organic Agriculture (FiBL), Ackerstrasse 113, Box 219, 5070 Frick, Switzerland; 3 Federal Research Centre for Cultivated Plants, Institute for Breeding Research On Agricultural Crops, Julius Kuehn-Institute (JKI), Rudolf Schick Platz 3a, 18190 Groß Lüsewitz, Germany; 4 Bavarian State Research Center for Agriculture (LfL), Institute for Crop Science and Plant Breeding, Kleeberg 14, 94099 Ruhstorf a. d. Rott, Germany

## Abstract

White lupin (*Lupinus albus* L.) is a high-protein grain legume alternative to soybean in Central Europe, but its cultivation is risky due to the fungal disease anthracnose that can cause severe yield damage. In addition, management of seed alkaloids is critical for human nutrition and animal feed. We report on a white lupin collection of genebank accessions, advanced breeding lines and cultivars that was genotyped and phenotypically characterized for anthracnose resistance and seed alkaloids and protein levels. Using genotyping by sequencing (GBS), SeqSNP-targeted GBS, BiomarkX genotyping and Sanger sequencing, a genetic resource of genome-wide SNPs for white lupin was established. We determined anthracnose resistance in two years field trials at four locations with infection rows and measured seed alkaloids and protein levels by near-infrared spectroscopy (NIRS). Few white lupin breeding lines showed anthracnose resistance comparable or better than Celina and Frieda, currently the best commercial cultivars in Germany. NIRS estimates for seed alkaloids and protein levels revealed variation in the white lupin collection. Using genome-wide association studies (GWAS), we identified SNPs significantly associated with anthracnose resistance in the field representing known and new genomic regions. We confirmed the *pauper* locus and detected new SNP markers significantly associated with seed alkaloids. For the first time, we present loci associated with total grain protein content. Finally, we tested the potential of genomic prediction (GP) in predicting the phenotype of these three quantitative traits. Application of results and resources are discussed in the context of fostering breeding programs for white lupin.

**Supplementary Information:**

The online version contains supplementary material available at 10.1007/s00122-024-04665-2.

## Introduction

Food security and soil fertility can be significantly improved in Europe by increasing domestic legume cultivation for feed and food (Foyer et al. 2016). Developments in the EU Common Agricultural Policy and national policies have called attention toward increasing domestic plant-based protein production and, in particular, to so far underutilized grain legumes. Overall EU soya bean production is expected to increase by 33% in the period 2022–2032, and at the same time, given an expanding area and increasing yields, the production of pulses is projected to increase by 2.4 million t and to reach 6.7 million t in 2032 (EU 2022). According to the medium-term outlook for EU agricultural markets, the EU will import less legumes and may become largely self-sufficient in the upcoming 10 years. Among underutilized grain legumes, white lupin (*Lupinus albus* L.) has gained attention as alternative to soybean given its comparable protein content (Prusinski 2017), potential health benefits (Bähr et al. 2014), suitability for sustainable production and acceptability to consumers (Lucas et al. 2015). In particular, white lupin as cool-season species from the Mediterranean region is tolerant to frost in early developmental stages as well as to summer drought during maturation. Because of this, it is a good spring-sown alternative to soybean in Central European conditions with cooler climate in spring.

The seeds of white lupin contain 33–47% protein on a dry weight basis (Pereira et al. 2022; Erbaş et al. 2005) with an acceptable level of essential amino acids (Prusinski 2017). While the composition and properties of white lupin proteins have been investigated in several studies (Bähr et al. 2014; Pereira et al. 2022), little is known about the genetic determination of protein content. In addition, lupins are a good source of other nutrients, such as lipids, dietary fiber, minerals and vitamins (Pereira et al. 2022). Beside the positive nutritional aspects, the presence of toxic quinolizidine alkaloids provides a bitter taste to lupin grain and limits its use (Frick et al. 2017). The alkaloid level is influenced by genetic factors as well as biotic and abiotic stressors (Rodés-Bachs and van der Fels-Klerx 2023). Nine recessive mutations have been identified in white lupin causing low-alkaloid accumulation, namely *pauper*, *primus*, *tercius*, *exiguus*, *nutricius*, *mitis*, *suavis*, *reductus*, and *minutus* (Hackbarth 1957, 1961; Porsche 1963; Satovic 1993; Troll 1958). The alleles *suavis* and *minutus* are incompletely described, while *primus* and *tertius* have been clearly identified as synonyms of *pauper*, which represents the most effective mutation in reducing alkaloid levels (Harrison and Williams 1982). *Pauper* has been widely used in white lupin breeding to produce cultivars with low-alkaloid content, so-called "sweet," and, to a lesser extent, *nutricius* and *exiguus*. The low-alkaloid mutations identified in white lupin drastically decrease the alkaloid production by the plant, but none of them eliminates it completely (Gustafsson and Gadd 1965; Gladstone 1970; Harrison and Williams 1982). Recent advances on the study of quinolizidine alkaloids biosynthesis in white lupin brought to the identification of the gene underlying the *pauper* locus and the detection of a single causal mutation SNP that leads to a biosynthesis pathway blockage (Mancinotti et al. 2022, 2023).

Another major limiting factor for lupin production is anthracnose, a seed- and airborne disease caused by the fungal pathogen *Colletotrichum lupini* (Talhinhas et al. 2016) which can lead to severe yield loss in areas with high disease pressure. Lupin anthracnose symptoms are characterized by a typical bending and twisting of stems and pods with necrosis with orange masses of conidia (Talhinhas et al. 2016). The use of resistant cultivars is economically and environmentally more efficient option compared to chemical seed disinfection or long-term storage (Alkemade et al. 2022a) and application of fungicides in the field, especially because currently there is no treatment option available that offers a guarantee of disease-free seed lots. Plant resistance to anthracnose varies between lupin species and between cultivars within species. Single dominant resistance genes have been identified in narrow-leafed lupin (*Lupinus angustifolius* L.) and yellow lupin (*L. luteus* L.). In narrow-leafed lupin, known resistance to anthracnose is controlled by *Lanr1* from cv. Tanjil, *AnMan* from cv. Mandelup (Yang et al. 2004, 2008), and *LanrBo* from the breeding line Bo7212 (Fischer et al. 2015). The homolog to *Lanr1* in cv. Core 98 (PI 385149) (Lichtin et al. 2020) and the resistance gene designated *Llur* from cv. Taper (Haase and Ruge-Wehling 2019) confer resistance to anthracnose in yellow lupin. Conversely, anthracnose resistance in white lupin is polygenic (Alkemade et al. 2022b). Upon world-wide outbreak of anthracnose from 1970s, a large screening effort was conducted for finding resistance sources in white lupin, assessing the resistance in wild types and landraces from across the globe, with the identification of very few, moderately resistant accessions useful in variety development (Adhikari et al. 2013, 2009; Valente et al. 2002). Despite the limited availability of resistance sources in white lupin, the two cultivars Frieda and Celina have been released in Germany in 2019 showing moderate resistance against anthracnose in Central European climate conditions. These joined the handful of commercially varieties available for farmers in Europe. Actually, while formal breeding began in Germany after World War I due to a need for high-protein grain legumes adapted to temperate conditions (Hondelmann 1984), efforts in white lupin breeding have been limited and scattered compared to major crops. The lack of modern breeding tools has hampered until recently the progress of white lupin improvement. The establishment of genetic maps, definition of first molecular markers for key traits (Phan et al. 2007; Yang et al. 2010; Rychel-Bielska et al. 2020; Rychel and Książkiewicz 2019; Alkemade et al. 2022b), application of genomic selection (Rychel-Bielska et al. 2020; Annicchiarico et al. 2019, 2023; Pecetti et al. 2023) and publication of the genome sequence for cultivar Amiga (2*n* = 50, 451 Mb) and variants from 39 accessions (Hufnagel et al. 2020, 2021) allow now to boost breeding research and marker development for this valuable yet neglected crop.

The content of quinolizidine alkaloids is environmental and year dependent for most varieties and often above the suggested thresholds for human and animal consumption (200 mg/kg and 500 mg/kg, respectively, according to Federal Institute for Risk Assessment Germany (2017), even in commercial varieties carrying the *pauper* mutation. This means that marker-assisted selection for the causal *pauper* mutation cannot lead alone to the development of cultivars with very low and stable alkaloid content. More knowledge is needed about the other genetic determinants of alkaloids accumulation. Regarding anthracnose resistance, first molecular markers have been established by biparental QTL studies (Yang et al. 2010; Rychel-Bielska et al. 2020) and by a genome-wide association study (Alkemade et al. 2022b). Unfortunately, identified sources of anthracnose tolerance in white lupin in one environment appear inefficient in another, making it challenging to implement selection in practice. Adhikari et al. (2009) could not confirm, under Australian field conditions, the resistance in germplasm from Portugal, Spain and South Africa found by Valente et al. (2002) but identified Ethiopian landraces as resistant under Western Australian field conditions. The resistance of these Ethiopian landraces could not be confirmed by climate chamber tests and field trials in Switzerland (Alkemade et al. 2021). To our knowledge, no QTL has been published yet for total protein accumulation in the grain of white lupin.

Against this background, our study aims to improve knowledge for the genetic make-up of anthracnose resistance, seed alkaloids and protein levels in white lupin. In our study, we constituted a genetically broad collection of white lupin accessions and determined their anthracnose resistance in two years field trials with infection rows at four locations and two developmental stages and measured their seed alkaloids and protein levels by near-infrared spectroscopy. Genotyping by sequencing was applied to establish a first genetic resource of genome-wide SNPs for our white lupin (LUpin White, LUW) panel. This genotyping resource was enlarged by trait-specific SNPs combining known QTLs and recently published genome sources. The main objectives of our study were to assess the prediction ability of genome enabled models, determine highly relevant genomic regions by GWAS and deliver molecular markers to boost practical breeding programs in white lupin for anthracnose resistance, seed alkaloid and protein content.

## Material and methods

### LUpin white (LUW) panel

For this study, we have put together a white lupin collection (LUW panel) comprising 167 accessions ordered from the Genebank of the Leibniz Institute of Plant Genetics and Crop Plant Research (IPK, Gatersleben, Germany) and the USDA ARS National Plant Germplasm System. Accessions from genebanks have different stages of improvement and originate from Mediterranean countries, Eastern European countries, Germany, Chile, Ethiopia and Portugal. The LUW panel is completed by 73 advanced breeding lines from the Landwirtschaftliche Lehranstalten Triesdorf (LLT, Germany) and 15 cultivars, thus comprising a total of 255 white lupin accessions (Tab. S1). Winter accessions and wild accessions were not integrated in the LUW panel. Fourteen accessions of the LUW panel overlap with the pangenome panel by Hufnagel et al. (2021), namely Amiga, Dieta, Energy, Feodora, Figaro, Gyulatanya, Hansa, Kalina, Kiev Mutant, Nährquell, Neuland, Neutra, Start and Volodia.

### Phenotyping

Field trials were conducted at four sites in Germany, namely Gross Luesewitz (54° 4′ 35.12" N 12° 22′ 40.409" E), Triesdorf (49° 12′ 5.864" N 10° 39′ 8.802" E), Frankendorf (50° 58′ 5.753" N 11° 27′ 0.362" E) and Ruhstorf (48° 26′ 26.88" N 13° 20′ 9.55" E). White lupin germplasm was screened for anthracnose resistance at the four experimental sites in the years 2020 and 2021 in a randomized complete block design and two replications of each accession. Seeds were sown in 1.00–1.35-m rows with every third row sown to cultivar Amiga, containing *Colletotrichum lupini* infected seeds as disease spreader rows. Anthracnose severity was assessed at the three critical developmental stages BBCH38 (juvenile stage), BBCH63 (flowering) and BBCH77 (green ripe) using a 1–9 scale where 1 is without symptoms and 9 is extremely damaged or dead with strong, typical symptoms.

Seeds were propagated in Triesdorf, Ruhstorf and Gross Luesewitz for field experiments. Near-infrared spectroscopy (NIRS) on seeds propagated at Triesdorf under pollinator-free condition (tunnel) was performed before sowing with a PERTEN DA7250 NIR analyzer (PerkinElmer GmbH) to estimate alkaloid and protein content in the grain (% the dry matter). Non-destructive measurements on 200-g grain samples were repeated twice for each sample to obtain an average value. The alkaloid and protein contents are estimated based on LLT in-house calibration established on 150 wet laboratory-quantified samples at the University of Heidelberg (Institute of Pharmacy and Molecular Biotechnology). The alkaloid and protein data were collected for 172 accessions only, because the other samples didn’t reach the minimum required amount of 200 g. Of these, 166 were used for GWAS and GP.

### Genotyping

DNA isolation from seeds and subsequent genotyping by sequencing (GBS) of all samples in one batch was conducted at LGC Genomics GmbH (Berlin, Germany). DNA samples were isolated from the original seed samples received from the suppliers. Half seed per accession was put into 2-mL tubes, cut into smaller pieces and crushed with 5-mm steel ball in the Geno/Grinder® (Spex SamplePrep, Metuchen, USA). Lysis was performed for 1 h at 60 °C with lysis buffer PVP and 30µL proteinase K (20 mg/mL). DNA isolation was followed with magnetic beads using sbeadex livestock kit (LGC Genomics, Berlin, Germany) on KingFisher Flex System (Thermofisher, Darmstadt, Germany). DNA samples were digested with restriction enzyme *ApeKI* and sequenced with Illumina NextSeq 500/550 system. GBS reads were analyzed according to company's protocols and using *L. albus* genome version 1.0 as reference sequence (Hufnagel et al. 2020), downloaded from https://whitelupin.fr/. White lupin genome coverage of GBS reads was determined using coverage function from samtools version 1.11 and *L. albus* reference genome version 1.0.

By using published variants in the 39 accessions of the white lupin pangenome (Hufnagel et al. 2021), 96 additional SNPs (Tab. S2) in eight candidate genes along the *pauper* locus were selected and 80 have been successfully genotyped in the LUW panel using BiomarkX platform (Standard BioTools™). To proceed with SNP enrichment, literature-supported genomic regions for anthracnose resistance in European cultivated lupin species were selected, namely ALB02 and ALB04 in white lupin (Rychel-Bielska et al. 2020), syntenic region of narrow-leafed lupin resistance locus *Lanr1* (Yang et al. 2012) on Lalb_Chr10 and syntenic region of yellow lupin resistance locus sca82074 (Lichtin et al. 2020) on Lalb_Chr04. BLAST analysis was performed to identify syntenic regions between white lupin and narrow-leafed lupin and yellow lupin, respectively. A total of 1,826 variants (Tab. S3) for the identified genomic regions relevant to anthracnose tolerance were enriched in the white lupin germplasm by SeqSNP-targeted GBS at LGC Genomics GmbH (Berlin, Germany) using the same batch of DNA samples used for GBS.

Re-sequencing (Sanger sequencing) of the candidate gene *Lalb_Chr05g_0216161*, putatively involved in anthracnose resistance response (Alkemade et al. 2022a), in the white lupin germplasm was done at Biosearch™ Technologies (Berlin, Germany). For the Sanger sequencing, genomic DNA was isolated from leaf tissue of three-week-old plants according to Plaschke et al. (1995). Four overlapping fragments (Tab. S4) were amplified by PCR in 20ul reaction volume with 0.8uM of each primer, 0.2 mM of each dNTP, 1.5 mM MgCl_2_, 0.1 unit Go Taq G2 Flexi Taq Polymerase (Promega GmbH, Walldorf, Germany) and 30 ng genomic DNA and cycling (BioRad thermocycler) with one cycle at 96 °C initial denaturation for 5 min, 35 cycles with 96 °C for 30 s, 55 °C annealing for 60 s and 72 °C for 60 s and finally one cycle with 72 °C for 10 min. Both strands of each fragment were re-sequenced on the ABI3730xl DNA Analyzer system (Applied Biosystems) at Biosearch™ Technologies (Berlin, Germany). We used Sequencher™ program version 5.4.6 (Gene Codes Cooperation, Ann Arbor MI, USA) for sequence alignment and editing. Amplicon sequences were aligned by ClustalW (Thompson et al. 1994). The alignment was manually corrected using BioEdit software (Hall 1999). All positions given in the text correspond to the genome positions of the white lupin genome reference assembly, version 1.0 (Hufnagel et al. 2020).

### Data analysis

In addition to the anthracnose phenotype measurements at BBCH63 and BBCH77 in the field, we calculated the average infection level across these two developmental stages to interpolate the infection development. Outliers were removed using Rosner test to adjust for normality of the data with a maximal suspected outlier per genotype (across year and location) of two at a type one error rate of 0.01. We used the Cullis approach to calculate the heritability. Subsequently, adjusted means across years and environments for the anthracnose infection were calculated by the formula:

1 $${Y}_{gye} = \mu + {g}_{i} + {e}_{k} + {y}_{j } + \left({{e}_{k} x {y}_{j} x b}_{i} x {r}_{i}\right) + \left({e}_{k} x {y}_{j} x {i}_{g} \right) + \varepsilon$$

where *Y* represents the anthracnose phenotype, *g* the genotype, *i* the genotype’s replicates, *k* the collection of *e* environments and *j* both examined years *y.* The block *b* x row *r* of each replicate *i* was used as random factor, nested in year and environment. Lastly, the replicate *i* of each genotype *g* was nested in environment *e* and year *y.* $\varepsilon$ indicates the residual error. Random factors are indicated by brackets.

Besides, we also calculated the adjusted means for each year and environment, separately using the formula

2 $${Y}_{g} = \mu + {g}_{i} + \left({ b}_{i} x {r}_{i}\right) + \left( {i}_{g} \right) + \varepsilon$$

In the procedure of model selection g * e * y, interaction turned out to be not significant. Therefore, adjusted means separately for two years and three locations are presented to year and environment specific QTLs [Eq. 2] along QTLs across years and environments [Eq. 1]. Analog, adjusted means were calculated for the seed quality traits alkaloid and protein levels, using the formula:

3 $${Y}_{g} = \mu + {g}_{i} + \left({y}_{j}\right) + \varepsilon$$

A GWAS was performed on these adjusted means (Tab. S5) and 24,576 SNPs (22,627 SNPs from GBS analysis with read depth of 8, 1,826 SNPs from targeted GBS, 80 SNPs from BiomarkX genotyping and 43 SNPs from Sanger sequencing, Tab. S6) by running the multi-marker linear mixed model (MMLM), FarmCPU and BLINK algorithm in GAPIT 3.3.1 R package with the first three principal components as covariables (Wang and Zhang 2021; Liu et al. 2016; Huang et al. 2019). Significant markers detected based on a minor allele frequency (MAF) < 0.02 were considered low confidence. Further, missing genotyping information was not imputed. The threshold for a significant marker–trait association was set to *p* < 0.05/marker count (Bonferroni correction).

To obtain an overview across the examined set of genotypes, the kinship of the 255 genotypes was calculated using the "rrBLUP" R package (Endelman 2011), with a maximum missing genotyping rate per marker of 30% and a minimum MAF of 0.02 (13,222 SNPs used). The broad-sense genomic heritability (24,576 SNPs) was calculated using the "sommer" R package (Covarrubias-Pazaran 2016). The visualization of the kinship was performed using "ComplexHeatmap" (Gu et al. 2016). The LD decay calculations were extracted from the GAPIT GWAS analysis.

We combined the pheno- and genotypes in a PCA plot to better understand the interconnection and correlation among subgroups. We, therefore, assigned a high and low infection group based on the anthracnose scoring of five (below = low, above = high). Further, all markers with more than 30% missing genotyping information were excluded to minimize the effect of missing genotyping information, and the remaining missing marker information was imputed with the average across the genotypes. The PCA was constructed based on the genotypes (13,222 SNPs used), while the phenotype class was added as metadata. QQ plots and marker density plots were generated with "CMplots" R package (Yin et al. 2021). A heatmap of candidate loci regions was created using "LDheatmap" R package (Shin et al. 2006).

Lastly, a genomic prediction approach was performed by applying a ridge regression model, implemented in the R package "rrBLUP." Markers with more than 40% missing were excluded, and the remaining missing genotyping information was imputed with the average across all other genotypes for the respective marker (15,563 SNPs used). The cutoff was set to 40% to balance the reduction in markers with the error introduction by the imputation. We used a cross-validation approach for the genomic predictions, where five (seed traits) and ten (anthracnose) genotype subsets were created. The process was an iterative approach, where each genotype subset was used four (seed traits) or nine times (anthracnose) to train the model and one time to test it. We performed five cross-validations for the seed traits as only 166 genotypes were used, and the test set would become too small to make meaningful assessments of the prediction ability with smaller test groups. The correctness of the model fit was measured by the Pearson correlation metric comparing the measured phenotype with the predicted value in the test set. This process was repeated once using the entire marker set and once using only the detected QTLs of each trait. Subsequently, we compared the Pearson correlation scores across all cross-validations trait-wise between the whole marker set and the QTLs using a nonparametric Kruskal–Wallis test to check for significant performance differences between both sets of markers.

## Results

### SNP data

GBS of 255 white lupin accessions resulted, on average, in 3.2 Mio reads per sample, with a minimum and maximum of 826,741 reads (SmB050) and 7,162,426 reads (SmB176), respectively, a N50 of 3,598,788 reads and mapping rate of 93.4%. A total number of 22,627 SNPs having at least 8 reads per accession were further used in this study.

Sanger sequencing of the candidate gene *Lalb_Chr05g0216161* from Alkemade et al. (2022a) resulted in 3.700 bp covering 3′-UTR, first exon, intron, second exon and 5'-UTR. According to the reference genome, 184 bp are missing within the second exon in the alignment of the Sanger sequences. Sanger sequencing failed in 13 of the 255 accessions. The 43 SNPs detected in the remaining 242 accessions of the LUW panel resulted in three haplotypes with a frequency higher 5% (Hap1 = 60.41%, Hap2 = 11.43%, Hap3 = 5.31%) and 30 minor haplotypes with a frequency below 2%. We detected 20 SNPs, all with frequency below 1%, that were not already present in the white lupin pangenome panel. The remaining 23 SNPs were also identified by Hufnagel et al. (2021) in the panel of 39 white lupin accessions. The position of the two SNPs associated with anthracnose resistance in Alkemade et al. (2022a) was confirmed.

Using SNPs from GBS analysis, candidate gene sequencing and alkaloid content phenotypic data, a pilot GWAS was calculated (data not shown). The analysis did not reveal statistically significant SNPs for the main alkaloid locus *pauper* on chromosome Lalb_Chr18 described in literature. This was most likely due to insufficient marker density and balanced linkage phase between SNPs in white lupin (Hufnagel et al. 2021; Alkemade et al. 2022a, present data). To overcome this, the approach was to enrich SNPs with physical distance of ca. 2.000 bp in genomic regions of interest to increase GWAS statistical power. For alkaloid content, 96 SNPs in candidate genes along the *pauper* locus were selected from the published variants in the 39 accessions of the white lupin pangenome (Hufnagel et al. 2021), and 80 were successfully genotyped in our white lupin collection using BiomarkX platform (Standard BioTools™, Tab. S2). The array was compiled to genotype SNPs located in eight candidate genes along the *pauper* locus: *Lalb_Chr18g0051351*, *Lalb_Chr18g0051471*, *Lalb_Chr18g0051511*, *Lalb_Chr18g0051521*, *Lalb_Chr18g0051531*, *Lalb_Chr18g0051541*, *Lalb_Chr18g0051551*, *Lalb_Chr18g0051561*. Seven SNPs of the 96 SNPs on the BiomarkX array technically failed. In addition, all nine SNPs in the gene *Lalb_Chr18g0051471* present on the BiomarkX array showed no amplification, which might indicate wrong assembly in the reference genome. The remaining 80 SNPs increased SNP density from 1SNP/12.2 kb (GBS data only) to 1SNP/753 bp (GBS data + BiomarkX data) within candidate genes along the *pauper* locus. For anthracnose resistance, significantly associated loci in pilot GWAS as well as literature-supported genomic regions for anthracnose resistance in lupins were selected, namely ALB02 and ALB04 in white lupin (Rychel-Bielska et al. 2020), syntenic region of narrow-leafed lupin *Lanr1* (Yang et al. 2012) on Lalb_Chr10 and syntenic region of yellow lupin sca82074 (Lichtin et al. 2020) on Lalb_Chr04. Between 227 and 671 SNPs (1,826 in total, Tab. S3) from variants in the white lupin pangenome panel were compiled for nine genomic regions of 80–1300 kb in size reaching an average physical SNP distance between 1975 bp and 2007 bp. These SNPs were genotyped in the white lupin germplasm by SeqSNP-targeted GBS. SeqSNP-targeted GBS resulted in 191,413,914 sequencing reads, translating to an average of 446,186 reads per sample and an average effective target SNP coverage of 219x. A total set of 1826 SNPs was extracted for further analysis.

In summary, we generated a genotypic resource for a panel of 255 white lupin accessions consisting of 24,576 SNPs (22,627 SNPs from GBS, 1826 SNPs from targeted GBS, 80 SNPs from BiomarkX genotyping and 43 SNPs from Sanger sequencing, Tab. S6) covering all 25 chromosomes with, on average, 1SNP/65 kb and, in particular, candidate regions for anthracnose resistance and seed alkaloids, 1SNP/2.150 bp (Fig. S1).

### Diversity array

Based on the SNPs from GBS, we established, for practical breeding purposes, an array with 96 SNPs distributed along the 25 white lupin chromosomes based on the BiomarkX platform (Standard BioTools™). This diversity array can be used to (i) perform crossing control of F1 plants, (ii) determine purity of seed batches and (iii) roughly characterize diversity in new white lupin materials introduced in the breeding program. The selected 96 SNPs occur in at least 167 accessions of the LUW panel, are covered by 8 reads per accession and have a missing rate below 20% and a MAF between 0.4 and 0.5, to allow for a balanced detection of both SNP alleles (Tab. S7). The frequency of heterozygous SNP calls for the selected SNPs was below 0.5% in our white lupin germplasm. The suitability of this genotyping array has already been proved in own breeding programs from the Bavarian State Research Center for Agriculture (LfL) and the Research Institute of Organic Agriculture (FiBL, data not shown).

### Population structure, kinship and linkage disequilibrium (LD)

**Fig. 1:**
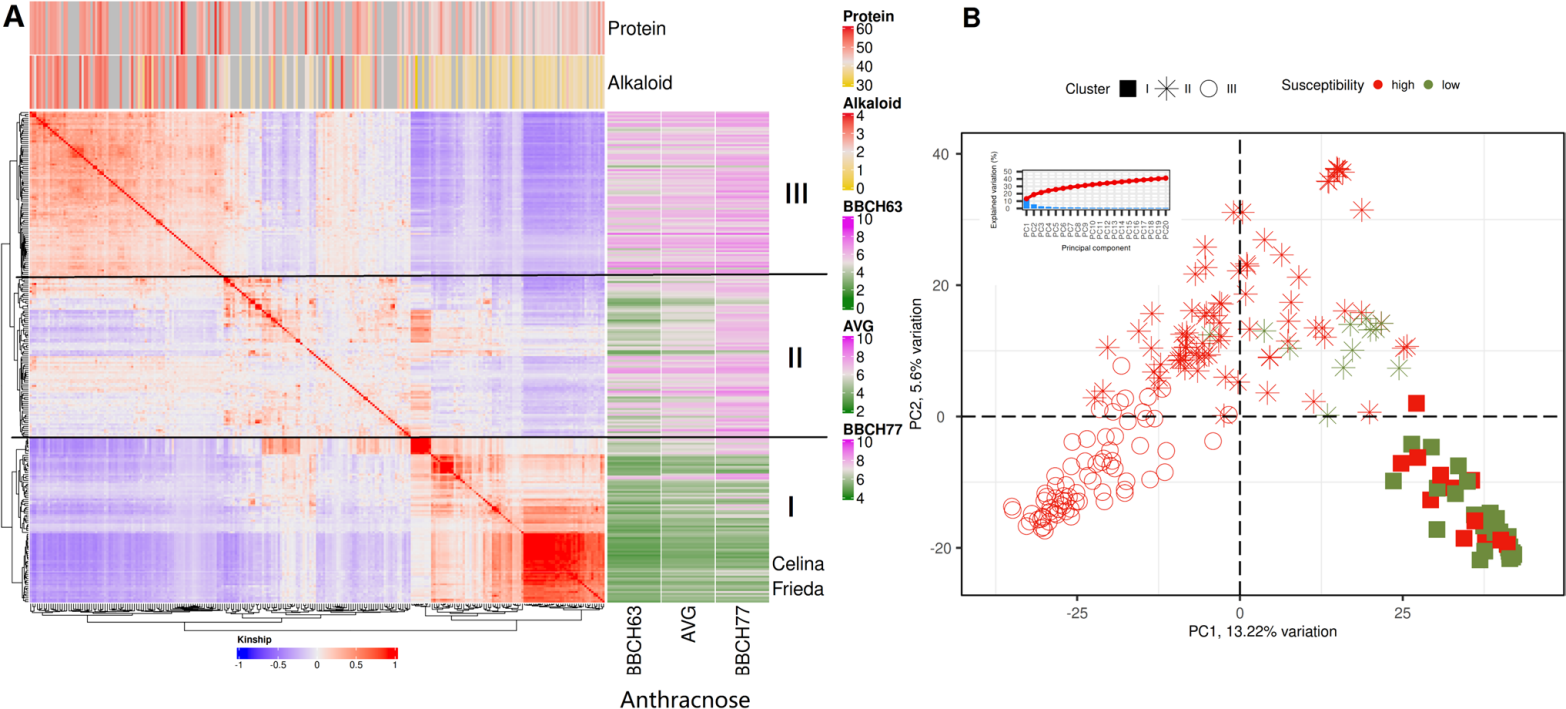
Genetic characterization of the white lupin LUW collection. **(A)** Kinship matrix of the LUW panel with cluster tree (left, bottom) and corresponding phenotypes for anthracnose (right) and seed quality (top). The color code for kinship, anthracnose infection and seed quality traits is given in the legend. The kinship is clustered in three groups of related genotypes **(B)** principal component (PCA) analysis based on genotyping data. Boxes, stars and circles illustrate the same clusters as presented in sub figure A. Red and green color of boxes, circles and stars indicate high and low anthracnose susceptibility, respectively. The explained variation in the first 20 PCs (blue bars) and the cumulated explained variation (red curve) are shown in the small graph

In the present study, a white lupin collection of cultivars, advanced breeding lines and genebank accessions with different geographical origins was compiled (LUW panel). The cluster analysis (Fig. 1A) based on genotypic data indicated three groups separating first the advanced LLT breeding lines (group I) from the other accessions (groups II and III). In group I, the cultivars Celina and Frieda and 41 of 73 LLT advanced breeding lines are found. The groups II and III suggest a split according to geographical origin, with group II being assigned mostly to accessions from Germany and France and group III including the accessions with origins from Hungary, Spain, Italy and Greece. The kinship analysis (Fig. 1A) revealed a clear separation between LLT advanced breeding lines and the other accessions in the LUW panel with weak relationship between accessions in group I with those in II and III. The relation of accessions in group I is higher with those in group II (especially with breeding lines and cultivars) compared to group III. Within group I, the breeding lines cluster according to their genetic ancestry and are strongly related. This shows the strength of the diversity bottleneck in white lupin, especially when considering material from a single breeding program (Fig. 1B). Within group II, the cultivars Amiga and Feodora, the old cultivar Neutra and three breeding lines reportedly resulting from a crossing with Neutra showed strong relatedness.

**Fig. 2:**
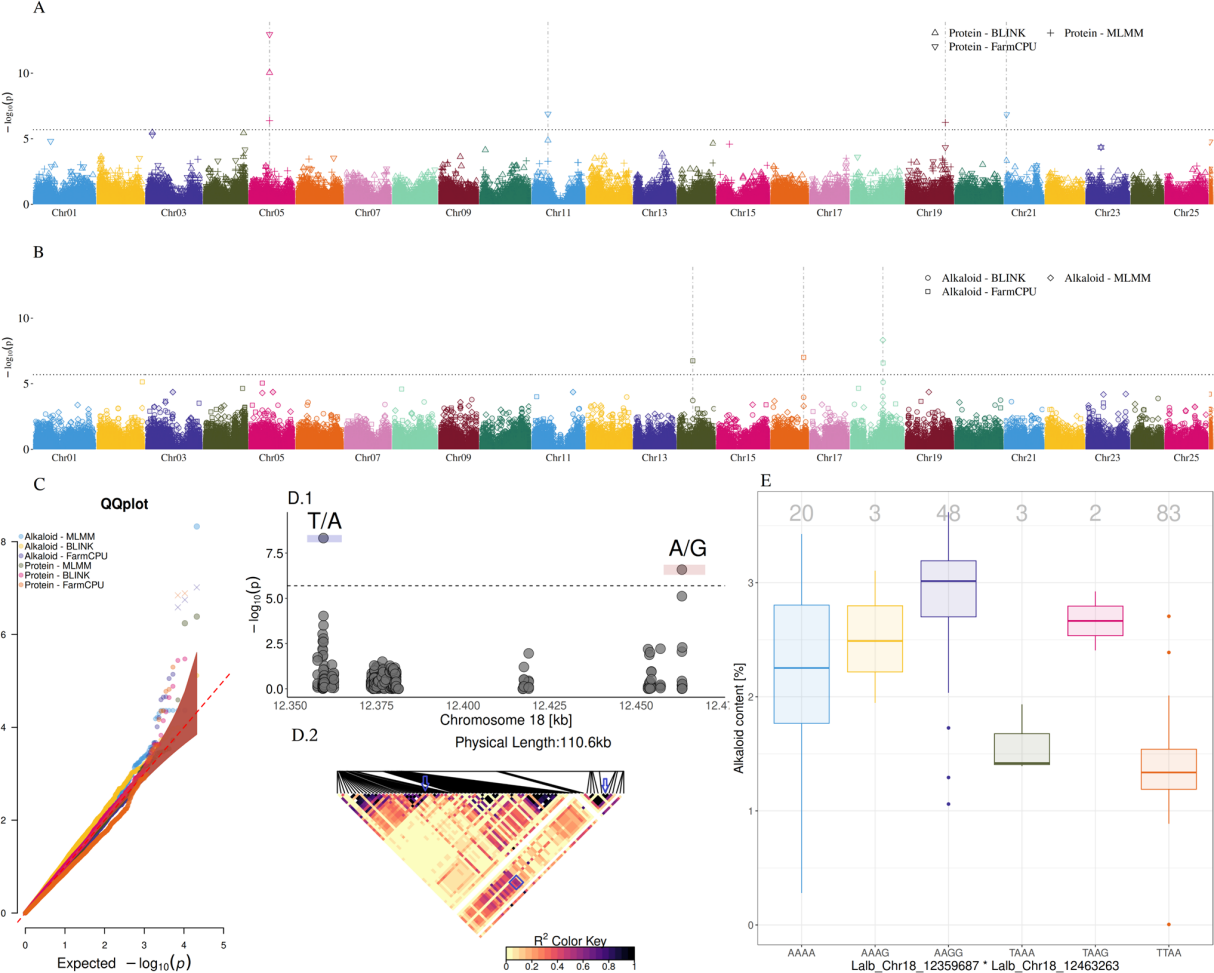
Results of genome-wide association study for seed protein and alkaloid content. **(A)** Manhattan plot for protein and **(B)** alkaloid content with **(C)** corresponding Q–Q plots by different statistical approaches (see text) showing SNP association with seed quality traits. Statistical approaches are shown in different symbols and chromosomes are shown in different colors. The dotted horizontal line indicates the -logP significance threshold of 5.7. Vertical dashed lines indicate QTLs. **(D.1)** Section of *pauper* locus on chromosome Lalb_Chr18 with significantly associated SNPs, along with **(D.2)** a SNP-wise linkage disequilibrium plot among all genotyped SNPs along the *pauper* locus. (**E**) Boxplot for the alkaloid seed content for every haplotype derived from an allele combination of both QTLs detected in the *pauper* region. Gray numbers above the boxplots illustrate the sample size for each haplotype. The NIRS seed alkaloid measurements were adjusted by subtracting the measured alkaloid content by the lowest (negative) measured value and adding an offset value of 0.005

LD between SNPs in the white lupin panel was low and decayed below *r*^*2*^ = 0.1 within 1 kb (Fig. S2) considering the available markers across the 25 chromosomes all together. In addition, LD observed between SNPs in genes along the *pauper* locus, the re-sequenced candidate gene *Lalb_Chr05g0216161* and the SNP enriched genomic regions (data not shown) was, in average, low, except for specific genomic loci, such as the two significant SNPs for alkaloid content on Lalb_Chr18 (Fig. 2D.2).

### Anthracnose resistance in the field

Field testing for anthracnose resistance was conducted in 2020 and 2021 at four locations in Germany with two replicates each and scoring at three developmental stages—juvenile (BBCH38), flowering (BBCH63) and “green ripe” maturity when ca 75% pods have reached full size (BBCH77). Disease symptoms were only observed in exceptional cases during juvenile stage BBCH38, and therefore, the related data were not used in the analysis. The infection increased during plant development and peaked at green ripe. Characteristic symptoms such as twisting of main shoot, side shoots and pods, lesions with orange conidial mass and dying of the entire plant were observed. No accession showed full resistance against the fungal attack. Scoring data showed a skewed distribution toward high values at the end of the growing season. At Triesdorf, no variation could be observed at BBCH77; thus, this location was excluded from further analyses. While at the other locations the infection was still moderate at flowering (mean = 4.4), at green ripe stage most accessions showed massive symptoms (mean = 7.2, Fig. S3A). On average, LLT breeding lines revealed better resistance scores (51 accessions out of 73 have an average score below 4.5) compared to the investigated genebank accessions that were very heavily damaged by the disease. Our field trials confirmed the moderate resistance of cultivars Celina and Frieda, with mean values of BBCH63 = 2.5 and 3.2 and BBCH77 = 5.6 and 5.8, respectively. High broad sense heritability was observed with *H*^*2*^ = 0.74 (BBCH63) and *H*^*2*^ = 0.77 (BBCH77), respectively. Variance analysis revealed a significant influence of accessions, environment and year on trait variation (Tab. S8). The strongest effect on the anthracnose infection levels was determined by the accession, reaching up to 43% of the total variance explained. The environment explained less than 2% of the variance, while the year effect was highest in the BBCH63 stage (12%). The replicate and position in the field did not contribute on average more than 1% of explained variance. These two terms were included in the mixed model as random factors to generate the adjusted means for each accession.

**Fig. 3:**
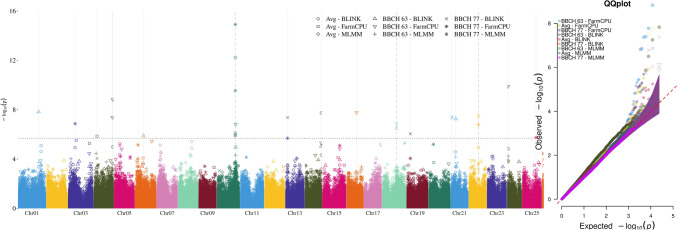
Manhattan (left) and corresponding Q–Q plot (right) by different statistical approaches (see text) showing SNP association with anthracnose resistance at three different developmental stages. Statistical approaches are shown in different symbols and chromosomes are shown in different colors. The dotted horizontal line indicates the -logP significance threshold of 5.7

We performed two complementary GWAS to identify marker–anthracnose associations. For the first, we used the adjusted means across all years and environments to identify stable QTLs across all experimental sites. Applying multi-marker mixed linear model, FarmCPU and BLINK models, we identified 17 unique QTLs across 14 different chromosomes (Table 1, Fig. 3). The most significant QTL for anthracnose resistance was detected on chromosome Lalb_Chr10 at position 16, which could explain 61% of the total phenotypic variance in all three stages (BBCH63, BBCH77, average), and all three applied GWAS approaches. The second highest QTL is located on chromosome Lalb_Chr24 at position 395 kb. Though explaining up to 61% of the phenotypic variance, it could only be detected for the BBCH63 stage by the FarmCPU model. Besides, the third most significant QTL was detected on chromosome Lalb_Chr04 at position 16 Mb, explaining 38% of the phenotypic variance. Four further QTLs were characterized by an explained variance above 30%, while the remaining show *r*^*2*^ values below 10%.

**Table 1:** Significant SNP associations for the traits anthracnose resistance and seed quality

Trait		Locus							
	Phenotype	Chromosome	Position	MAF	−logP	r^2^	Effect	N	Confidence
Anthracnose resistance	Average	Lalb_Chr04	2′315′230	0.41	*5.86*	35.40	−0.16	*255*	High
		Lalb_Chr14	14′436′450	0.36	*7.74*	31.98	−0.19		High
		Lalb_Chr22^a^	8′112′890	0.44	*7.51*	3.18	0.46		Low
	BBCH 63	Lalb_Chr01	17′758′245	0.40	*7.83*	9.85	−0.36		Low
		Lalb_Chr04	16′424′332	0.26	*7.38*	6.61	0.32		Low
		Lalb_Chr04	16′717′968	0.38	*8.83*	37.65	0.29		High
		Lalb_Chr06	7′022′154	0.21	*5.87*	3.18	−0.34		Low
		Lalb_Chr16	8′530′965	0.17	*7.78*	9.85	0.47		Low
		Lalb_Chr18	12′358′001	0.47	*6.85*	2.48	0.26		Low
		Lalb_Chr21	182′950	0.15	*7.40*	6.61	1.07		Low
		Lalb_Chr21	3′907′702	0.15	*7.25*	37.65	−1.11		High
		Lalb_Chr24	394′166	0.17	*9.89*	60.93	−0.64		High
	BBCH 77	Lalb_Chr03	5′487′444	0.01	*6.87*	2.48	1.01		Low
		Lalb_Chr10^b^	16′065′293	0.27	*14.91*	60.93	−0.37		High
		Lalb_Chr13	1′366′208	0.45	*7.37*	5.94	−0.20		Low
		Lalb_Chr19	2′669′672	0.11	*6.05*	35.40	−0.50		High
		Lalb_Chr25	12′010′870	0.08	*5.75*	5.94	−0.25		Low
Seed quality	Alkaloid	Lalb_Chr14	5′832′122	0.30	*6.75*	53.30	−0.26	*166*	High
		Lalb_Chr16	12′866′499	0.06	*7.01*	3.03	0.61		Low
		Lalb_Chr18	12′359′687	0.48	*8.33*	59.09	−0.47		High
		Lalb_Chr18	12′463′263	0.34	*6.58*	54.39	0.21		High
	Protein	Lalb_Chr05	7′755′912	0.01	*12.96*	9.61	4.94		Low
		Lalb_Chr11	5′705′330	0.08	*6.89*	20.51	−1.18		High
		Lalb_Chr19	15′634′884	0.05	*6.24*	18.78	−2.95		High
		Lalb_Chr21	297′544	0.39	*6.84*	27.37	−0.98		High

For each locus chromosome and genomic position in bp, minor allele frequency (MAF), −log(P) value, explained variance (*r*^*2*^), effect on trait, sample size (N) and SNP confidence are given. Low-confidence SNPs are shown in italic

Only highest QTL peak reported

Found for^a^

Average & BBCH 77 ^b^ BBCH 63, Average & BBCH 77

Further, a GWAS was conducted on the adjusted means for each of the three environments and two years separately, resulting in six additional GWAS per BBCH stage. The previously reported locus on Lalb_Chr10 at 16 Mb was again observed at all developmental stages in three environments (Frankendorf, Gross Luesewitz, Ruhstorf) and years (2020, 2021) (Fig. S4, Tab. S9). Besides, a QTL on chromosome Lalb_Chr08 at position 12 Mb was detected at BBCH63 and average in two environments (Gross Luesewitz, Ruhstorf) in the year 2020 (2021 only Ruhstorf). Further minor QTLs on fifteen other chromosomes, especially at BBCH77, were only detected in a single year and environment.

### Seed alkaloids and protein content

To determine seed alkaloids and protein content, we used the non-destructive NIRS method to further use the seeds for field experiments. In the 172 accessions of the LUW panel phenotyped for alkaloid and protein content, a wide variation was observed for seed alkaloid and protein content (Tab. S8, Fig. S3B), mainly caused by genebank accessions. Trait heritabilities were high with *H*^*2*^ = 0.83 for seed alkaloids and moderate with *H*^*2*^ = 0.63 for protein content in the grain, respectively. Variance analysis revealed a significant influence of accessions and year on both traits (Tab. S8). The genotype effect was with 88% and 65% explained variance 17.6 × and 2.6 × bigger than the year effect for seed alkaloids and protein content, respectively.

For seed alkaloids, GWAS revealed significantly associated SNPs on chromosomes Lalb_Chr14 and Lalb_Chr16 in addition to the *pauper* locus on Lalb_Chr18 (Table 1, Fig. 2B). While all three GWAS algorithms detected two associated SNPs in the *pauper* region, only the FarmCPU detected the two additional QTLs (Fig. 2B and C). The two QTLs in the *pauper* region are 103,576 bp apart and are described by a moderate linkage of *r*^*2*^ = 0.56. (Fig. 2D.1 and D.2). The haplotype combinations of these two SNPs highlighted that the allele combinations AAAA, AAAG and AAGG were associated with a significantly increased seed alkaloid content compared to the TTAA haplotype (*p* < 0.01, Fig. 2E).

For seed protein content, SNP associations were identified on chromosomes Lalb_Chr05, Lalb_Chr11, Lalb_Chr19 and Lalb_Chr21 (Table 1, Fig. 2A and C). Although the QTL on chromosome Lalb_Chr05 is the most significant association with a -logP value of 12.96 and detected by all three models (MLMM, FarmCPU, BLINK), the explained variance is below 10%, mainly affected by the low MAF of 0.01 (Table 1). The QTLs on chromosomes Lalb_Chr11 and Lalb_Chr21 were detected by FarmCPU model and explained 21% and 27% of the phenotypic variance, respectively. The fourth QTL associated with seed protein content was detected by MLMM model on chromosome Lalb_Chr19 and explained 19% of the total phenotypic variance.

### Genomic prediction

**Fig. 4:**
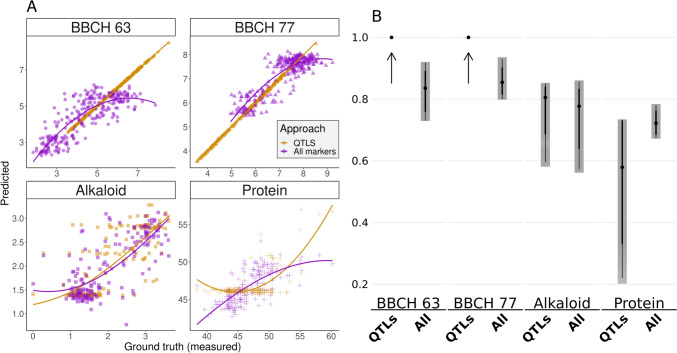
Genomic prediction for anthracnose and seed quality in the white lupin LUW collection. **(A)** Measured versus predicted values and corresponding regression lines for anthracnose infection at two developmental stages as well as seed alkaloid level and protein content using all SNP markers (purple) and only significantly associated SNP markers (QTLs, yellow). **(B)** Pearson correlation prediction for all traits using the two marker sets. The dots give the average prediction value from the cross-validation, while the gray bars show the confidence interval; the arrows indicate the dot when the confidence interval is zero

Although we detected SNPs for all three traits explaining up to 61% of the total variance, anthracnose resistance, seed alkaloids and protein content are quantitative traits. Multiple genes interact to form the final phenotype. Therefore, multiple markers are needed to illustrate the quantitative trait architecture. To account for this limitation, we performed a cross-validated genomic prediction for the four traits anthracnose resistance at BBCH63, BBCH77, seed protein and seed alkaloid content. Genomic predictions have been performed using (i) the entire set of imputed SNPs with less than 40% missing genotyping information (15,563 markers) and (ii) a second set comprising only the significantly associated SNP markers (QTLs) for each trait. High average predictive abilities of 0.81 and 0.85 were reached for anthracnose resistance BBCH63 and BBCH77 stages for the full marker model, respectively. For anthracnose resistance, predictive abilities were significantly improved when using only the associated SNPs (BBCH63 and BBCH77, *r*^*2*^ = 0.999) compared to the entire set of SNPs (*p* = 0.0002, Fig. 4). For both seed-related traits, no significant differences in the prediction ability were observed when using only QTLs in the genomic prediction model compared to the full marker model (*p* > 0.05, Fig. 4). Compared to the anthracnose, the general prediction levels were decreased to an average of *r*^*2*^ = 0.75 for the seed alkaloid and *r*^*2*^ = 0.65 for seed protein content.

## Discussion

### LUpin white LUW panel as resource for breeding

In the present study, a collection (LUW panel) of white lupin (*Lupinus albus* L.) cultivars, advanced breeding lines and genebank accessions with different geographical origins was compiled to identify resistance sources against the fungal disease anthracnose and for studying two key quality traits, i.e., seed alkaloids and protein content. In our field trials, we could confirm the moderate anthracnose resistance of the cultivars Celina and Frieda. In the LUW panel, we could not identify genetic resources with resistance level against anthracnose comparable or better than these two cultivars. While in a GWAS study based on 181 accessions collected from the center of domestication and traditional cultivation regions, Alkemade et al. (2022a) reported a high resistance of the cultivar BLU 25 phenotyped under controlled conditions with a stem-wound inoculation protocol, we could not confirm this phenotype in our field trials. We speculate that observing different responses to anthracnose for the same accession in different environments and growing conditions might be either due to *C. lupini* strains with different pathogenicity or due to impure seed batches resulting in variable response. Additionally, the white lupin resistance mechanisms may be different across plant developmental stages. Alkemade et al. (2022a) worked in controlled conditions with inoculation of 2-weeks old seedlings, meaning that the cultivar BLU 25 might show a good resistance in the first growing stages but not at flowering and maturity as of scoring in our field trials. Alike, we could not confirm the association with increased resistance for the candidate gene on Lalb_Chr05, *Lalb_Chr05g0216161*, which might indicate different resistance response of the plant depending on developmental stage and/or fungal strain.

In the cluster analysis based on genotypic data, three groups emerged showing a separation into the very strongly related advanced LLT breeding material, including the released cultivars Celina and Frieda (I), and other accessions (II and III). The genetic relatedness of genotypes within group is very high in group I. Accessions in group III are more related to each other compared to the ones in group II. The groups II and III suggest a split according to geographical origin. Hufnagel et al. (2021) assembled a set of 39 white lupins consisting of modern cultivars, landraces and wild accessions from 17 countries. Their cluster analysis identified five groups describing early-flowering spring accessions from Germany and France (type 1) and Poland (type 2), winter accessions (type 3), Iberian and Appenine Peninsula accessions (type 4) and wild accessions (type 5). A similar grouping can also be observed in the LUW panel, with group II being assigned to accessions from Germany and France (cf. type 1) and group III mainly to accessions from Hungary, Spain, Italy and Greece (cf. type 2 and type 4). Winter accessions and wild accessions were not integrated in the LUW panel. Grouping according to geographic origin was also found in a set of 200 white lupin accessions collected from the Mediterranean region, Atlantic islands, East Africa, Europe, Chile and Australia (Alkemade et al. 2022b), with an overlap of observed geographic groups. This mixing of origins in geographic groups was also observed in the LUW panel.

The linkage disequilibrium (LD) between SNPs in the LUW panel was low and decayed within 1 Kb (*r*^*2*^ = 0.1). This rapid genome-wide LD decay was confirmed in other white lupin studies (Alkemade et al. 2022b; Hufnagel et al. 2021). Determination of genetic structure (relationship and cluster analysis) and linkage disequilibrium of the LUW panel were used for genetic characterization but are also important aspects for the subsequent GWAS. The rapid LD decay requires high marker density and prompted us to proceed with SNP enrichment in genomic regions of interest to increase the statistical power of the association study. While this approach helps to confirm already reported QTLs, the statistical power of the GWAS in so far unknown regions is still limited by the sub-optimal SNP distribution and low SNP density obtained by GBS. The use of the same or improved phenotypic data with a strongly enlarged SNP set, e.g., from whole genome sequencing, would allow to overcome this limitation of our study.

The PCA combining phenotypes and genotypes highlighted how resistance to anthracnose in the LUW panel is highly correlated with the subgroups, with low infection values for the advanced LLT breeding lines of group I and high infection values for the accessions in groups II and III. The population structure in the LUW panel and, in particular, the same genetic background of the advanced LLT breeding lines and a resulting segregating kinship are confounding factors in a GWAS which we have addressed by using FarmCPU, and BLINK models additional to a multi-marker mixed model approach in GAPIT R. While FarmCPU reduces the effect of the strong clustering effect of the anthracnose resistance in the global kinship by recalculating a local kinship (FarmCPU) for each tested marker, BLINK uses the principal components to reduce false positive QTLs due to the population structure, and “iteratively incorporates associated markers as covariates for testing markers to eliminate their connection to the cryptic relationship among individuals” (Wang and Zhang 2021). The application of an MLM or GLM model was impractical with the given populations, as the GLM would produce too many false positive QTLs and the MLM underestimates the true count of QTLs due to the narrow relationship among individuals and their correlation with anthracnose infection levels.

In our study, heritability values are high for content of alkaloids in the seeds (*H*^*2*^ = 0.83) and anthracnose resistance (*H*^*2*^ = 0.77 at BBCH77). These values are comparable to other studies for seed alkaloids (Beyer et al. 2015b) and anthracnose resistance (Alkemade et al. 2022b). While no report is available for heritability of protein content in white lupin, a value of *H*^*2*^ = 0.82 is reported in narrow-leaved lupin by Beyer et al (2015a) which is higher compared to our study (*H*^*2*^ = 0.63). Based on the heritability values and the large variation identified for the three studied traits, the LUW panel provided a good basis for the identification of SNP markers for these traits.

### GWAS for anthracnose, seed alkaloids and protein content

GWAS in the LUW panel yielded significantly associated SNP markers for the major breeding traits anthracnose resistance, seed alkaloids and protein content in white lupin. In the GWAS, significant associations for 17 (across years and environments) and 23 (year and environment-wise) SNP markers with anthracnose resistance were identified, which include already identified and new QTL regions. The SNPs are distributed on 14 (across years and environments) and 17 (year and environment-wise) of the 25 white lupin chromosomes, confirming the quantitative architecture of anthracnose resistance in white lupin (Książkiewicz et al. 2017; Rychel-Bielska et al. 2020; Alkemade et al. 2022b). Several resistance loci have been previously identified in European lupin species (*L. albus*, *L. luteus*, and *L. angustifolius*) which were also found in the present study. The resistance locus ALB04 (Rychel-Bielska et al. 2020) could be confirmed with the association of two SNP markers (Lalb_Chr04_16,424,332 and Lalb_Chr04_16,717,968). The SNP markers localize in genes encoding a protein kinase of the RLK-Pelle-RLCK-IXb family (*Lalb_Chr04g0264281*) and a calcium-transporting ATPase (*Lalb_Chr04g0264661*), respectively, which were described to play critical roles in abiotic and biotic stress response (Park and Shin 2022; Gish and Clark 2011). Interestingly, *Lalb_Chr04g0264281* was also identified in white lupin mapping population study by Rychel-Bielska et al. (2020). The resistance locus *Lanr1* from narrow-leaved lupin (Yang et al. 2012) was anchored to chromosome Lalb_Chr10 in white lupin via sequence comparison in the present study. Enrichment of SNPs for this genomic region identified six significant SNPs for anthracnose resistance in the syntenic region on Lalb_Chr10 (0.7 Mb, position 15,801,130 to 16,501,391). Thus, the *Lanr1* resistance locus from narrow-leafed lupin (Yang et al. 2010, 2012), which has also been identified in yellow lupin (Lichtin et al. 2020), we could confirm in white lupin. Remaining significantly associated SNPs must be further investigated and validated by subsequent research studies. Additionally, we identified four significant SNP markers for seed alkaloid content. Our results on alkaloids should be interpreted in relation to the measurement method used for phenotyping this trait. The indirect estimation of alkaloid content by NIRS allows for clear separation of landraces with wild-type level of alkaloids from “sweet” modern breeding lines and varieties and allowed to observe a wide variation in the LUW panel. However, NIRS estimation implies a high technical detection threshold which makes it difficult to identify small differences among the “sweet” accessions. In another German research project, a calibration for non-destructive NIRS has also been established for *L. angustifolius* (German Federal Office for Agriculture and Food, agreement: 2814EPS009). Here it was concluded that the method can be used very well for screening, but the detection limit of the alkaloid content is ca. 500 μg/g dry matter (Fischer et al. 2018). Because of this, our phenotype dataset is not meant to determine associations for modifier genes that modulate the level of alkaloids in “sweet” varieties in addition to the major genes.

Regarding the major genetic determinants of alkaloid content, we could confirm the *pauper* locus on chromosome Lalb_Chr18 (Książkiewicz et al. 2017; Phan et al. 2007; Rychel and Książkiewicz 2019) and the causal SNP Lalb_Chr18_12359687 in the *pauper* gene *Lalb_Chr18g0051511* (Mancinotti et al. 2023). In literature, in addition to the *pauper* locus, the two loci *exiguus* and *nutricius* are described for low-alkaloid content in white lupin. These loci were introduced in the beginning of white lupin breeding with the cultivars Neuland (SmB213, *exiguus*, 1937) and Nährquell (SmB098, *nutricius*, 1949) (Harrison and Williams 1982, Rychel and Książkiewicz 2019). The genomic position of these two alkaloid loci has not yet been clarified. In the present study, additional SNPs for alkaloid content were identified on chromosomes Lalb_Chr14, Lalb_Chr16 and Lalb_Chr18. There is no annotated gene in the proximal region of the SNP on Lalb_Chr14. Lalb_Chr16_12866499 and Lalb_Chr18_12463263 localize to genes encoding a putative WD40-like family transcription factor (Lalb_Chr16g0390531) and a 3-hydroxyacyl-CoA dehydrogenase (Lalb_Chr18g0051561), respectively. Further research is needed to clarify if either these genes themselves or genes in LD with the identified SNPs play a role in alkaloid biosynthesis. Nevertheless, in addition to the *pauper* locus, we identified two genomic loci that could be candidates for other alkaloid mutants, such as *exiguus* and *nutricius*. Lalb_Chr18_12463263 could be a case of co-selection with the published *pauper* causal mutation, even though simple LD with Lalb_Chr18_12359687 cannot be excluded.

Another interesting trait for white lupin breeders, besides anthracnose resistance and seed alkaloids, is protein content. Lupin seeds have been recognized as valuable for both human and animal diet. They are high in protein (30–40%) and dietary fiber (45–50%), low in fat (6%) and with virtually no starch (Beyer et al. 2015a). For protein content, significantly associated SNPs were identified for the first time in white lupin. These new findings complement modern breeding programs that mainly focus yield stabilization, resistance to abiotic stresses, resistance to diseases (mainly to anthracnose) and late maturing (Abraham et al. 2019). In addition, improvement in lupin grain quality is requested to meet the food industry needs (Beyer et al. 2015a).

### Genomic prediction

Genomic prediction in white lupin has been shown to be successful for grain yield, drought tolerance, anthracnose resistance and a set of morphological traits (Annicchiarico et al. 2023, 2019; Pecetti et al. 2023; Rychel-Bielska et al. 2020). In this study, the approach of genomic selection was applied to predict anthracnose resistance, seed alkaloid and protein content phenotype in white lupin. The developed statistical models showed high predictive abilities for all traits studied, comparable or even higher than predictive abilities for anthracnose (Rychel-Bielska et al. 2020), grain yield (Annicchiarico et al. 2019), drought tolerance (Pecetti et al. 2023), winter survival, onset of flowering, pod fertility, individual seed weight, plant height, leaf size, mainstem proportion of seeds and number of leaves (Annicchiarico et al. 2020). Running the genomic predictions on the detected QTLs resulted in improved prediction abilities of anthracnose compared to the full marker model. Further, we could not detect a disadvantage using the same approach for the seed quality traits. The high rate of explained variance per genotype made a “feature pre-selection” of markers for the genomic prediction model reasonable. Although these QTLs were also included in the full marker model, limitations in the model fitting, e.g., by assigning each marker an effect (even when very small), can negatively impact the models fit. Therefore, we conclude that it can be a valuable approach to combine QTLs in a genomic prediction model to improve “marker-assisted selection”. This said, we must remark that the detected QTLs might be specific to the examined collection. Therefore, the prediction approach comes with limitations and might not work equally well outside the tested gene pool, where other QTLs influence the phenotype.

### Prospects for white lupin breeding

Recent scientific advances in white lupin research such as publication of white lupin reference genome and genome-wide variants (Hufnagel et al. 2020, 2021) or identification of the causal mutation in *pauper* alkaloid metabolism (Mancinotti et al. 2023) allow the development of breeding relevant molecular tools. In this study, two genotyping arrays on the BiomarkX platform (Standard BioTools™) were established. The diversity array consists of 96 SNP assays that are distributed along the 25 chromosomes and is useful for controlling crossing success, determining seed lot purity and roughly characterizing diversity in a new set of white lupine accessions.

Significantly associated SNP markers in the presented GWAS might be useful for marker-assisted selection in white lupin breeding programs. For this purpose, trait-associated SNPs have been transferred to a second genotyping array to ensure reproducibility of SNPs in different material assortments. These SNPs need to be validated in order to implement their use in practical breeding. As application of our results, array genotyping of unknown germplasms can be requested for any breeding and research application to LfL.

## Conclusion

The present study assembled a white lupin panel (LUW panel) that exhibits genetic and phenotypic diversity and for which extensive genome-wide SNP data and robust phenotypic data on major traits in white lupin breeding were collected. Significantly associated SNP markers confirmed one published anthracnose resistance QTL of white lupin, showed significant association in genomic region homologs known to be involved in this trait from yellow and narrow-leafed lupin research and identified new marker–trait associations for this trait. We confirmed the causal SNP marker for the sweetness locus *pauper* and identified additional SNP markers associated with seed alkaloid content. First SNP markers associated with protein content were presented. Additionally, genomic prediction showed great potential for white lupin breeding, especially predicting the phenotype of anthracnose. The resources established by this study can be used and directly applied to improve and expand white lupin breeding.

### Supplementary Information

Below is the link to the electronic supplementary material.

Supplementary file1 (PDF 678 kb)

Supplementary file2 (PDF 471 kb)

Supplementary file3 (PDF 275 kb)

Supplementary file4 (PDF 481 kb)

Supplementary file5 (XLSX 30 kb)

Supplementary file6 (XLSX 25 kb)

Supplementary file7 (XLSX 83 kb)

Supplementary file8 (XLSX 16 kb)

Supplementary file9 (XLSX 32 kb)

Supplementary file10 (XLSX 22538 kb)

Supplementary file11 (XLSX 24 kb)

Supplementary file12 (XLSX 20 kb)

Supplementary file13 (XLSX 20 kb)

## Data Availability

GBS and SeqSNP-targeted GBS data were uploaded to NCBI short read archive (project ID PRJNA1089354) and can be downloaded from https://www.ncbi.nlm.nih.gov/sra/PRJNA1089354. Genotype data of the LUW panel including SNPs from all genotyping analyses (24,576 SNPs) and phenotypic data are provided as Supplemental material.
