# Supplementary material for: Genome-wide association study revealed significant SNPs for anthracnose resistance, seed alkaloids and protein content in white lupin
Source: Theor Appl Genet. 2024 Jun 10;137(7):155. doi: 10.1007/s00122-024-04665-2 (PMC11164739; doi:10.1007/s00122-024-04665-2)
Supplement: Supplementary file 1 — Supplementary file1 (PDF 678 kb) [file 122_2024_4665_MOESM1_ESM.pdf]

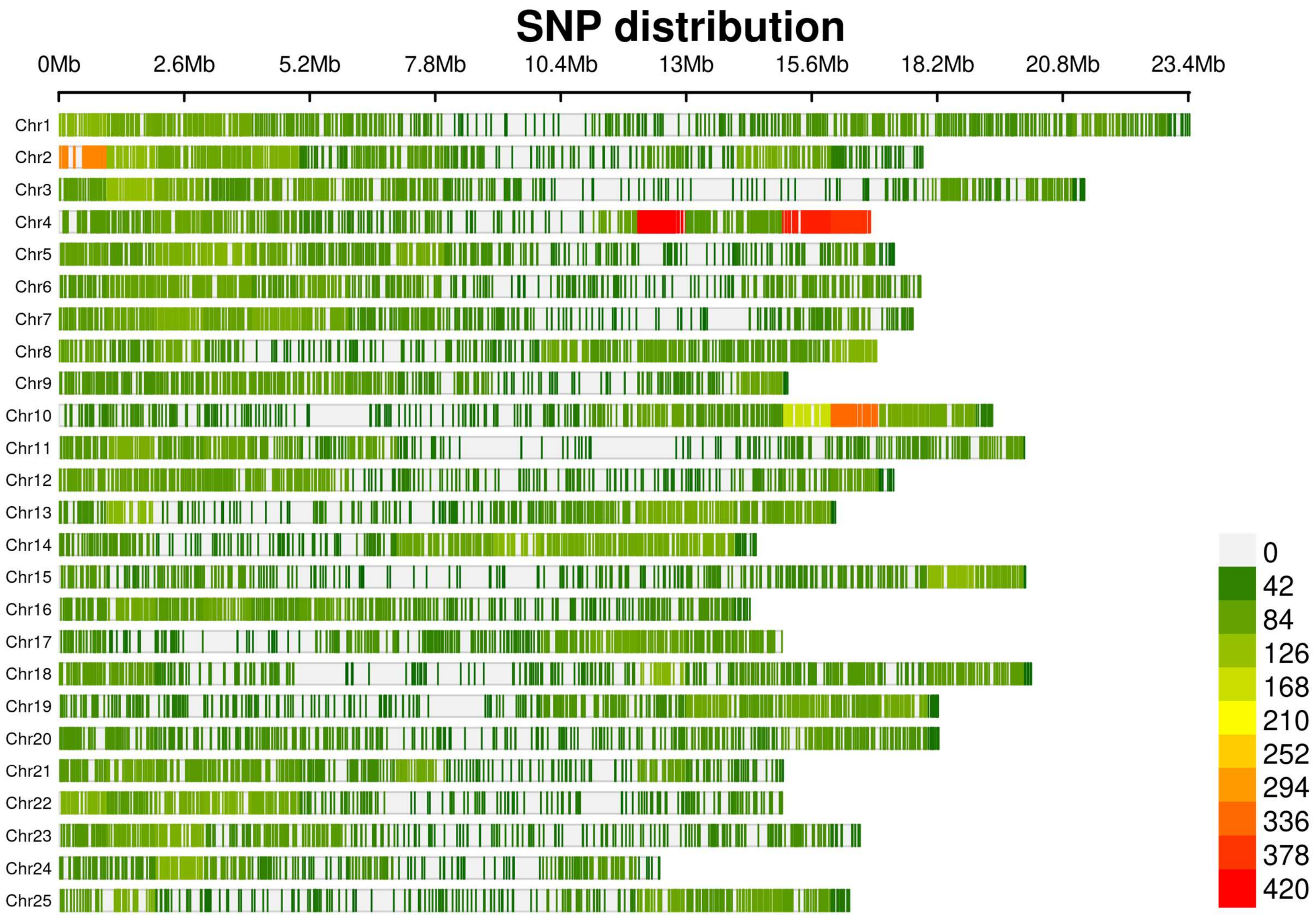

Figure S1. Distribution of 24,576 SNPs along the 25 white lupin chromosomes after genotyping-by-sequencing (GBS), SeqSNP target GBS, Standard Biotools™ genotyping and Sanger sequencing. The density is given as color code in SNP per kilobase.
