# Supplementary material for: Genome-wide association study revealed significant SNPs for anthracnose resistance, seed alkaloids and protein content in white lupin
Source: Theor Appl Genet. 2024 Jun 10;137(7):155. doi: 10.1007/s00122-024-04665-2 (PMC11164739; doi:10.1007/s00122-024-04665-2)
Supplement: Supplementary file 2 — Supplementary file2 (PDF 471 kb) [file 122_2024_4665_MOESM2_ESM.pdf]

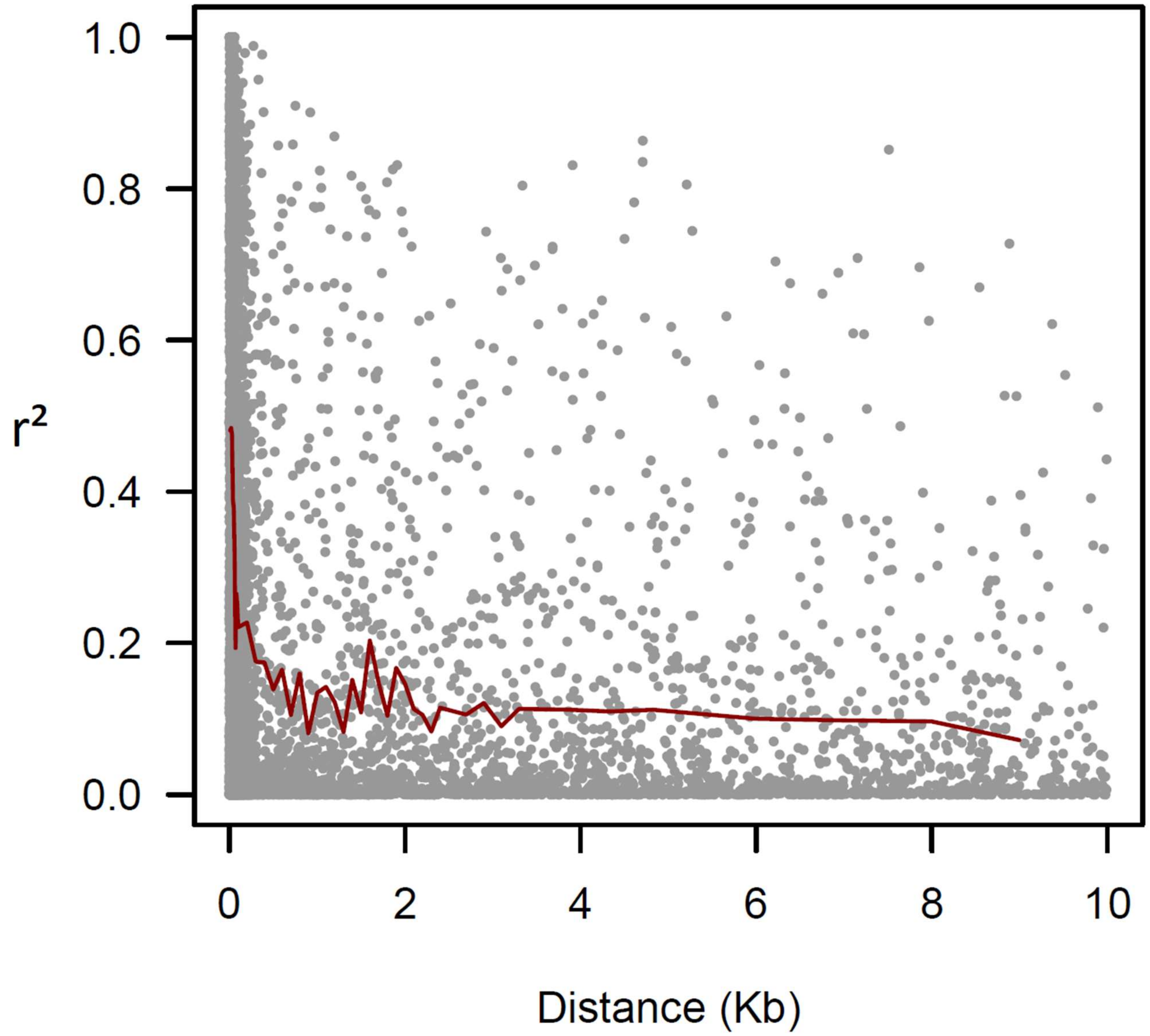

Figure S2. Genome wide linkage disequilibrium (LD) and LD decay curve of the white lupin collection. Estimates of LD between marker pairs were calculated as correlation (r^2) and plotted on physical distance shown in kilobases (Kb). LD decay with physical distance is indicated with a red line.
