# Supplementary material for: Genome-wide association study revealed significant SNPs for anthracnose resistance, seed alkaloids and protein content in white lupin
Source: Theor Appl Genet. 2024 Jun 10;137(7):155. doi: 10.1007/s00122-024-04665-2 (PMC11164739; doi:10.1007/s00122-024-04665-2)
Supplement: Supplementary file 3 — Supplementary file3 (PDF 275 kb) [file 122_2024_4665_MOESM3_ESM.pdf]

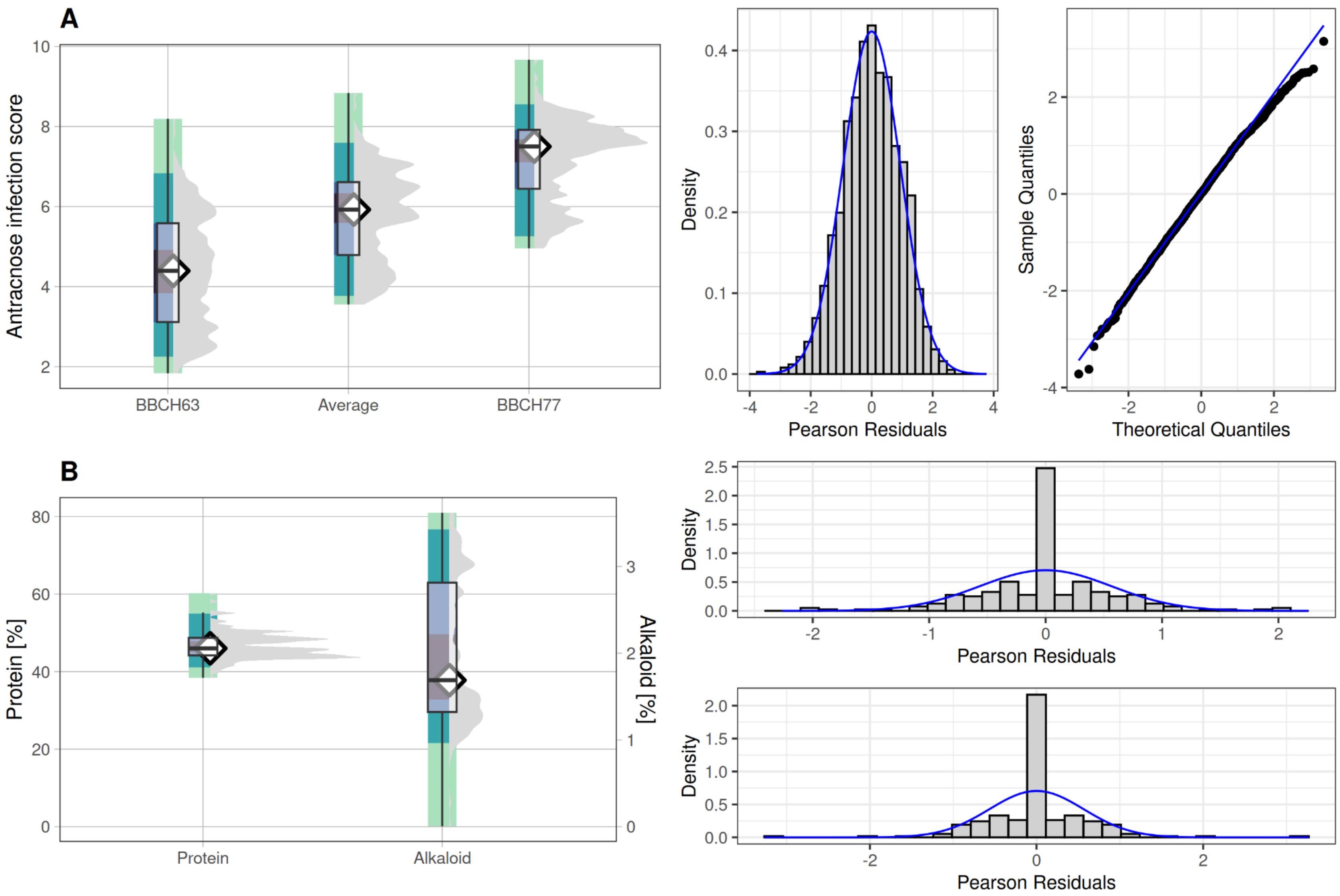

Figure S3. Adjusted phenotypic data for the traits anthracnose (A), protein and alkaloid (B). An off-set value of 0.005 was added to the raw NIRS values for alkaloids to avoid negative values. The colored bars (dark blue, blue, dark green, green) represent 25, 50, 95, and 100 % of the observations, while the mean is indicated as diamond. Boxplots with quartiles as well as density plots (grey) illustrate the data distribution. Residual and quantile distributions are shown on the right. The NIRS seed alkaloid measurements were adjusted by subtracting the measured alkaloid content by the lowest (negative) measured value and adding an offset value of 0.005.
