# Supplementary material for: Genome-wide association study revealed significant SNPs for anthracnose resistance, seed alkaloids and protein content in white lupin
Source: Theor Appl Genet. 2024 Jun 10;137(7):155. doi: 10.1007/s00122-024-04665-2 (PMC11164739; doi:10.1007/s00122-024-04665-2)
Supplement: Supplementary file 4 — Supplementary file4 (PDF 481 kb) [file 122_2024_4665_MOESM4_ESM.pdf]

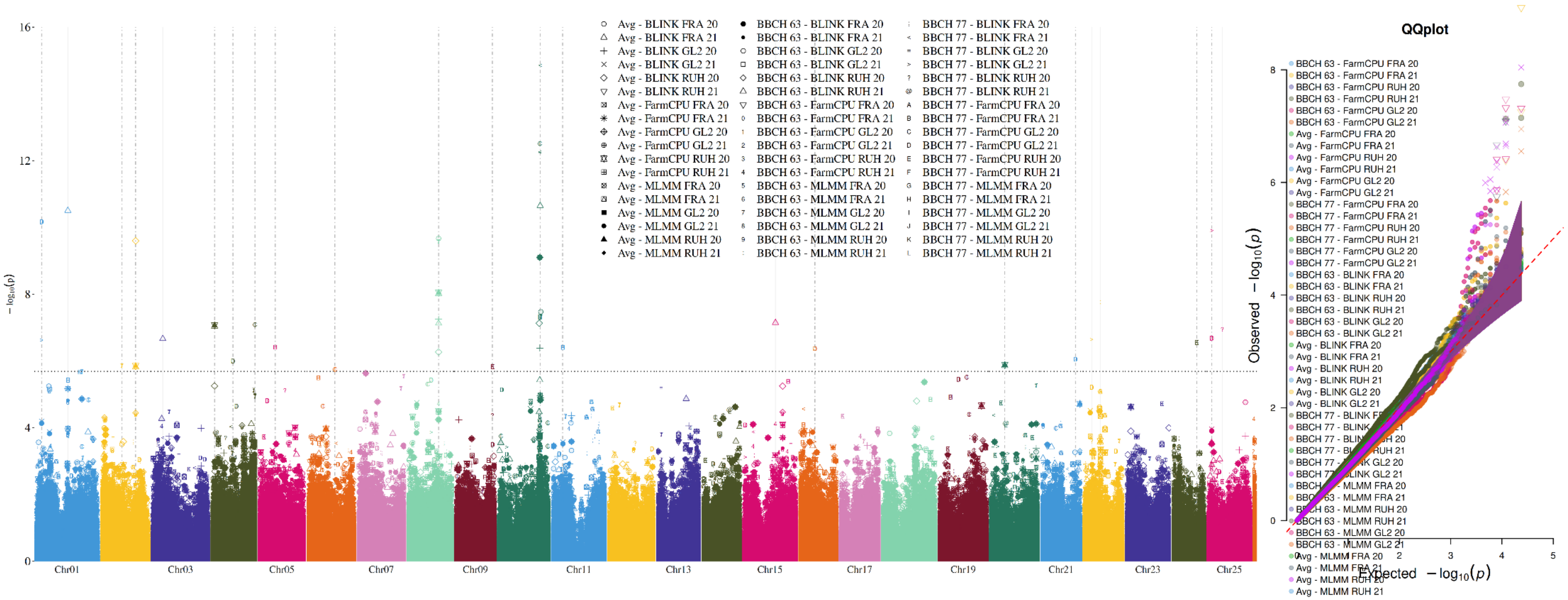

Figure S4. Manhattan (left) and corresponding Q–Q plot (right) by different statistical approaches showing SNP association with anthracnose resistance at three different developmental stages per environment and per year. Statistical approaches are indicated by different symbols and chromosomes are shown in different colors. The dotted line shows the fixed LOD threshold of 5.7 (P = 5.00E-05).
